# Active patient queue dynamics in a district hospital emergency centre: a cross-sectional analysis

**DOI:** 10.1016/j.afjem.2026.101021

**Published:** 2026-10-24

**Authors:** Mary Elizabeth Hoffe, Clint Hendrikse

**Affiliations:** Division of Emergency Medicine, University of Cape Town, Cape Town, South Africa

**Keywords:** Emergency centre, Active queue, Clinician workload, Patients per hour, Crowding, Low- and middle-income countries

## Abstract

**Introduction:**

Emergency Centre crowding is a persistent challenge, particularly in low- and middle-income countries, where resource constraints exacerbate mismatches between demand and capacity. Clinician productivity is central to EC flow and is typically measured using patients per hour (PPH), but this does not capture concurrent workload. The active queue (AQ), operationally defined as unresolved clinician-attributed patient episodes at any point during a shift, may complement PPH by capturing the concurrent nature of EC clinical work. This study aimed to describe AQ dynamics at a district-level EC and identify the clinician, shift, and flow factors associated with variation in queue size.

**Methods:**

A retrospective cross-sectional secondary analysis was conducted using data from 1 289 clinician shifts across three four-week periods in 2019 at a South African district-level EC. AQ was derived from timestamped patient flow data. Associations with clinician, shift, and system-level factors were assessed using one-way ANOVA and Spearman's rank correlation.

**Results:**

Median AQ was 1.50 (IQR 1.00–2.25), with a median peak AQ of 3.00 (IQR 2.00–4.00). AQ varied significantly by clinician category, employment type, and shift type (all p < 0.001), with higher values among senior clinicians and during night shifts. AQ remained stable at a median of 2.00 during the active working period before declining in the final shift quarter, while PPH declined progressively throughout. AQ and PPH were moderately correlated (rho = 0.43, p < 0.001). AQ was positively associated with patients waiting at shift start (rho = 0.19, p < 0.001) but not with patient acuity, despite acuity strongly suppressing PPH (rho = −0.44, p < 0.001).

**Conclusions:**

Emergency clinicians routinely manage concurrent patient loads, with AQ demonstrating a consistent mid-shift plateau. AQ complements PPH by capturing the concurrent dimension of clinician workload that throughput measures alone cannot reflect, with relevance to staffing and operational planning in district-level EC settings.

## African Relevance

• Emergency Centre (EC) crowding is compounded in many LMICs by limited resources, staffing shortages, and restricted access to inpatient care.
• Patients per hour measures how many patients a clinician completes, but not how many they are managing concurrently, a distinction with direct operational relevance in resource-constrained settings.
• This study provides the first locally derived description of concurrent clinician patient load dynamics at a district-level EC, offering an evidence base for staffing decisions and shift design that has previously relied on anecdote or high-income country benchmarks.
• Locally derived data on queue dynamics provide a foundation for evidence-based workforce planning and shift design in comparable settings.

## Introduction

Emergency centre (EC) crowding remains a global challenge affecting healthcare systems in both high-income countries (HICs) and low- and middle-income countries (LMICs), where resource constraints and inequitable workforce distribution amplify its consequences [1,2]. Crowding is driven by input, throughput, and output pressures, and is associated with delayed treatment, increased morbidity and mortality, and clinician burnout [[3], [4], [5]]. While input and output factors are often externally constrained, throughput, the domain of patient progression through the EC, remains most amenable to EC-driven improvement, with clinicians as its central drivers [4].

In South Africa, district-level ECs operate at the frontline of an overburdened health system, managing high patient volumes with limited access to diagnostics, inpatient beds, and specialist services [6,7]. Staffing models in LMIC ECs have historically relied on HIC benchmarks rather than locally derived evidence. Prior work from this EC described clinician output as substantially lower than HIC norms, and identified staffing category, shift type, and resource availability as key determinants of patients seen per hour (PPH) [8].

PPH remains the dominant measure of EC clinician output, but it captures throughput alone, how many patients a clinician completes per hour, and cannot describe the concurrent patient load managed at any point during a shift. Emergency clinicians do not work sequentially. They build an active queue of patients in various stages of assessment, investigation, and awaiting disposition simultaneously (Fig. 1). These concurrent demands are not reflected by PPH, yet are central to understanding how clinicians function and how workflow can be optimised [9]. Conceptually, the EC functions as a queuing system in which patients arrive at variable rates and are served by clinicians whose capacity is finite [10]. The AQ for a given clinician at time t can be expressed as:

AQ(t) = |{p: consultation_start(p) ≤ t < disposition(p)}|

where *p* denotes an individual patient episode, *t* is the point in time at which the queue is measured, and consultation_start(*p*) and disposition(*p*) represent the recorded timestamps for the start of consultation and the disposition decision respectively. This approximates Little's Law (L = λW), where L is the mean number in the system, λ the arrival rate, and W the mean time each patient remains open [11]. AQ may therefore reflect both the rate of opening consultations and downstream bottlenecks, e.g., investigation delays, admission waits, and referral processes, that prolong time to disposition [12]. Despite its relevance, the active patient queue, operationally defined as unresolved clinician-attributed patient episodes, has not been described or quantified in an LMIC district-level EC. This study aimed to describe the active patient queue at a district-level EC in Cape Town, South Africa, and to identify the clinician, shift, and flow factors associated with variation in queue size.

**Fig. 1:**
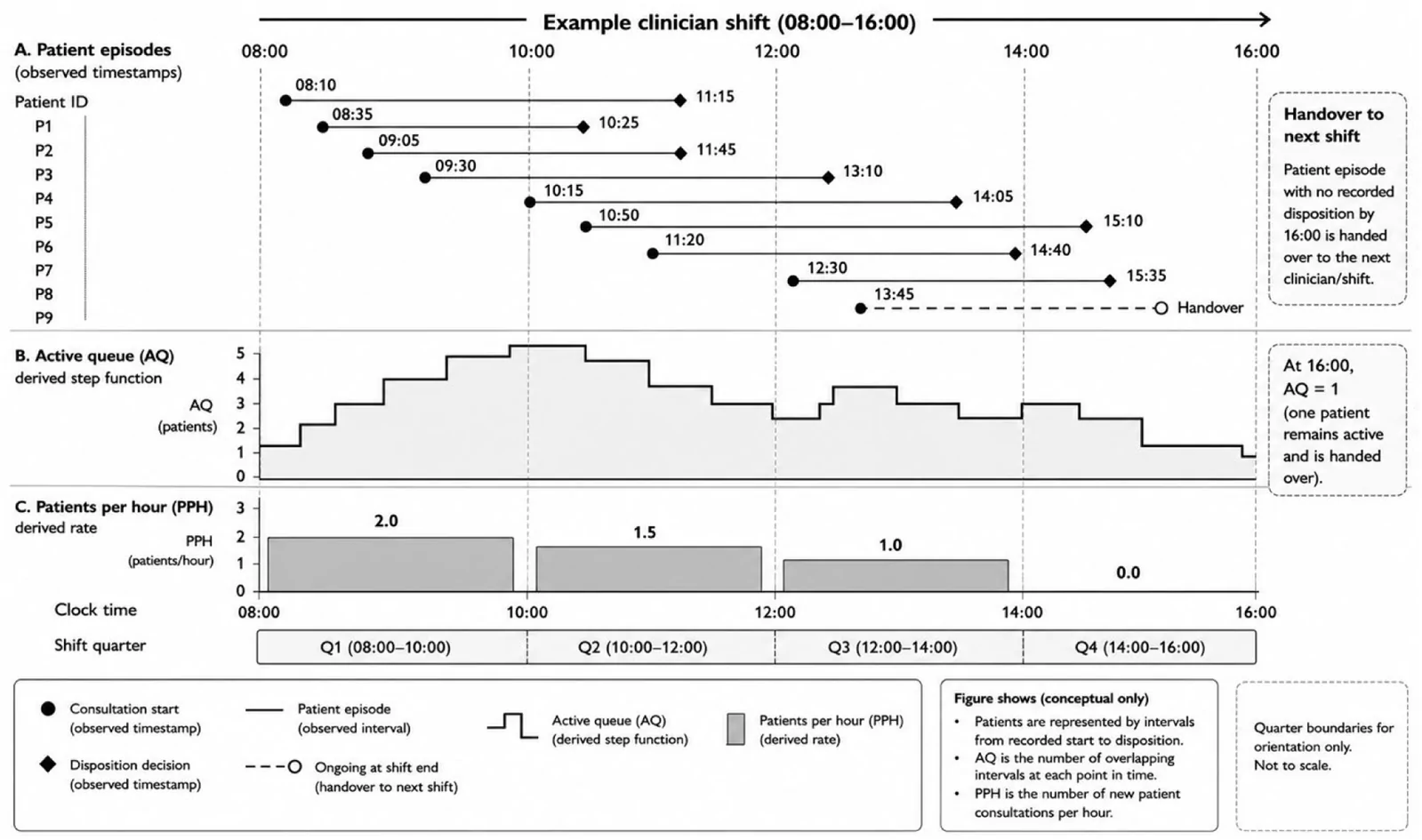
Conceptual illustration of active queue (AQ) and patients per hour (PPH) during a clinician shift. Each horizontal line represents a patient episode from recorded consultation start to recorded disposition decision. The AQ step function shows the number of active patient episodes at each point in time, while PPH represents the rate of new patient consultations initiated per hour. One patient episode remains unresolved at shift end and is handed over to the incoming clinician, illustrating how active patients contribute to shift handover. Quarter boundaries are shown for orientation only. Figure not to scale.

## Methods

### Study design

This was a retrospective cross-sectional secondary analysis of an existing dataset. The dataset comprised all clinician shifts across three randomly selected non-contiguous four-week periods in 2019 at the EC of Mitchells Plain Hospital. The original dataset, which informed a prior description of clinician output at this EC by Hoffe et al. [8], was reprocessed and expanded to derive the active queue (AQ) for each shift quarter and the number of new patients consulted per quarter.

### Study setting

This study was conducted at Mitchells Plain Hospital (MPH), a district-level hospital situated approximately 32 km from Cape Town, South Africa. MPH serves a predominantly low- to middle-income population of approximately 750 000–800 000 residents across Mitchells Plain and the adjacent Philippi township. The EC manages adult and paediatric patients in separate treatment areas, both staffed by emergency clinicians and supervised by specialist emergency physicians.

Clinicians are organised into teams of four, typically comprising a combination of interns (newly qualified clinicians in supervised practice), community service medical officers (CSMOs; junior doctors in their first year of unsupervised practice), medical officers, and emergency medicine registrars (specialist trainees). Teams rotate on a four-week cyclical roster. On weekdays, three shifts are scheduled per day; on weekends, two. One team is allocated per shift, covering both adult and paediatric areas. Additional locum clinicians and clinicians from other departments are periodically allocated to specific shifts.

### Study sample

All clinician shifts during three randomly selected four-week periods in 2019 were included. No formal sample size calculation was performed; all eligible shifts from the original dataset were utilised. Shifts with incomplete data were excluded. The original dataset comprised 1 291 clinician shifts; two (0.2%) were excluded due to incomplete data, yielding 1 289 shifts for the current analysis.

### Data collection procedure

De-identified data were extracted from HECTIS (Hospital and Emergency Centre Tracking Information System), an electronic patient tracking and registration database used across the Western Cape emergency centres. HECTIS records timestamped data for each patient encounter, including registration, triage, start of clinician consultation, disposition decision, and EC exit.

HECTIS is the official Western Cape Department of Health electronic patient tracking system used across public sector emergency centres. Data entry occurs in real time at multiple points during the patient journey: patient registration and pre-triage visibility are captured by administrative clerks on arrival; triage category and vital signs are entered by nursing staff; and clinician consultation, diagnosis, and disposition plan are entered by the responsible clinician. Physical exit from the EC is recorded as the final timestamp. Real-time data entry and built-in system checks support data quality throughout. HECTIS permits retrospective amendment of timestamps. Clinical event times are documented in the patient notes at the time they occur and subsequently entered into the system, meaning that retrospective entries reflect actual recorded clinical times rather than estimates. While this practice reduces systematic documentation delay bias, a degree of discrepancy between the precise moment of a clinical event and its recorded time remains inherent to any manually entered system and should be considered when interpreting AQ calculations. AQ in this study was derived from the clinician-entered consultation-start and disposition timestamps.

The AQ for each clinician was derived from these timestamps. For each shift quarter, the AQ was defined as the number of patients simultaneously under a clinician's active care - that is, the number of patients for whom a consultation had started but a disposition decision had not yet been recorded. Each shift was divided into four equal quarters based on total shift duration. The average AQ (AVQ) and maximum AQ per shift, and per shift quarter, were calculated from this reprocessed dataset alongside the previously derived PPH.

System-level flow metrics, including the number of patients present at shift start, new arrivals during the shift, and the average number of boarders, were also extracted and averaged per shift.

### Data analysis

Data were analysed using IBM SPSS Statistics, version 27. Categorical data are described using frequencies and proportions. Non-normally distributed continuous data are presented as medians and interquartile ranges (IQR). The correlation between AVQ and PPH was assessed using Spearman's rank correlation. Associations between AVQ and clinician, shift, and flow factors were assessed using one-way ANOVA. Spearman's rank correlations were also calculated between AQ and shift-level flow metrics, including patients waiting at shift start, new arrivals during the shift, mean total patients in the unit, mean boarders, and the proportion of high acuity patients per shift (emergent and very urgent triage categories combined). The association between AVQ and the number of consecutive shifts worked was assessed using one-way ANOVA. Statistical significance was defined as p < 0.05. AVQ and PPH were further categorised into quartiles for graphical depiction.

### Ethical considerations

Ethical approval for the original data collection was obtained from the Stellenbosch University Health Research Ethics Committee (HREC S20/01/021) and facility approval through the National Health Research Database (WC_202006_041). Ethical approval for the reprocessing of the dataset for the current analysis was obtained from the University of Cape Town Human Research Ethics Committee (HREC REF 337/2024). The dataset was confirmed to be de-identified prior to analysis.

## Results

A total of 1 289 clinician shifts involving 9 668 patient consultations by 111 clinicians were analysed. Senior clinician shifts (registrars and medical officers combined) accounted for 55% of all shifts, and the core EC team primarily employed at MPH EC comprised 85% of shifts. The remaining 15% were performed by locum doctors or clinicians from other departments. Forty-two percent of all shifts occurred outside of office hours (weeknights or weekends). The overall median PPH was 0.70 (IQR 0.50–1.00), consistent with the prior description from this EC [8]. Shift characteristics and AQ by category are summarised in Table 1.

**Table 1:** Shift characteristics, active queue and clinician output by category (n = 1 289).

	**n (%)**	**Average AQ, median (IQR)**	**Peak AQ,** **median (IQR)**	**PPH,** **median (IQR)**	**p**
**Overall**	1 289	1.50 (1.00–2.25)	3.00 (2.00–4.00)	0.70 (0.50–1.00)	
**Clinician category**					<0.001
Registrar	189 (15)	1.75 (1.25–2.50)	3.00 (2.00–4.00)	0.70 (0.50–0.92)	
Medical officer	511 (40)	1.75 (1.25–2.75)	3.00 (2.00–4.00)	0.80 (0.60–1.10)	
Community service MO	397 (31)	1.50 (1.00–2.25)	3.00 (2.00–3.00)	0.70 (0.50–1.10)	
Intern	192 (15)	1.00 (0.50–1.50)	2.00 (1.00–3.00)	0.38 (0.25–0.52)	
**Employment category**					<0.001
EC department	1090 (85)	1.50 (1.00–2.25)	3.00 (2.00–4.00)	0.70 (0.50–1.00)	
Locum	128 (10)	2.00 (1.44–3.50)	4.00 (2.00–5.00)	0.88 (0.62–1.10)	
Other department	71 (6)	0.75 (0.25–1.50)	2.00 (1.00–3.00)	0.38 (0.25–0.69)	
**Shift type**					<0.001
Weekday morning	398 (31)	1.50 (1.00–2.00)	3.00 (2.00–4.00)	0.60 (0.40–0.90)	
Weekday afternoon	350 (27)	1.50 (1.00–2.00)	2.00 (2.00–3.00)	0.62 (0.38–0.88)	
Weeknight	254 (20)	2.00 (1.50–2.75)	3.00 (2.00–4.00)	0.90 (0.60–1.20)	
Weekend day	167 (13)	1.50 (1.00–2.25)	3.00 (2.00–4.00)	0.67 (0.42–1.00)	
Weekend night	120 (9)	2.00 (1.50–3.00)	3.00 (2.00–5.00)	0.92 (0.67–1.10)	
**Shift length**					0.001
8 h	481 (37)	1.50 (1.00–2.00)	2.00 (2.00–3.00)	0.62 (0.38–1.00)	
10 h	506 (39)	1.75 (1.00–2.25)	3.00 (2.00–4.00)	0.70 (0.50–1.00)	
12 h	288 (22)	1.75 (1.25–2.50)	3.00 (2.00–4.00)	0.75 (0.50–1.08)	

AQ, active queue; PPH, patients per hour; CSMO, community service medical officer; IQR, interquartile range.

p values from one-way ANOVA across subcategories within each group.

The overall median AVQ was 1.50 (IQR 1.00–2.25) and the median peak AQ per shift was 3.00 (IQR 2.00–4.00). Significant variation in AVQ was observed across all categories (all p < 0.001), with the exception of shift length (p = 0.001). Among clinician categories, registrars and medical officers carried the highest median AVQ (1.75 for both), while interns had the lowest (1.00, IQR 0.50–1.50). Locum doctors had the highest median AVQ of any group (2.00, IQR 1.44–3.50) and the highest peak AQ (4.00, IQR 2.00–5.00), while clinicians from other departments had the lowest on both measures. Weeknight and weekend night shifts were associated with higher AVQs (median 2.00) compared to daytime and weekday afternoon shifts (median 1.50). Longer shifts were associated with modestly higher AVQs, with 10- and 12-hour shifts recording a median AVQ of 1.75 compared to 1.50 for 8-hour shifts. AVQ showed a modest increase with consecutive shift number. Median AVQ was 1.50 on first and early consecutive shifts, rising to 2.00 (IQR 1.50–2.75) for clinicians working more than six consecutive shifts (p = 0.0002).

AVQ and PPH followed divergent patterns across shift quarters (Fig. 2). PPH was highest in quarter one (median 1.00, IQR 0.50–1.50) and declined progressively to a median of 0.33 (IQR 0.00–0.67) in quarter four. AVQ remained stable at a median of 2.00 across quarters one to three before declining to 1.00 (IQR 0.00–2.00) in quarter four as consultations were completed towards shift end. In 820 shifts (63.6%), at least one patient remained under active care at the end of quarter four and was handed over to the incoming team. AVQ and PPH showed a moderate positive correlation across all shifts (Spearman's rho = 0.43, p < 0.001).

**Fig. 2:**
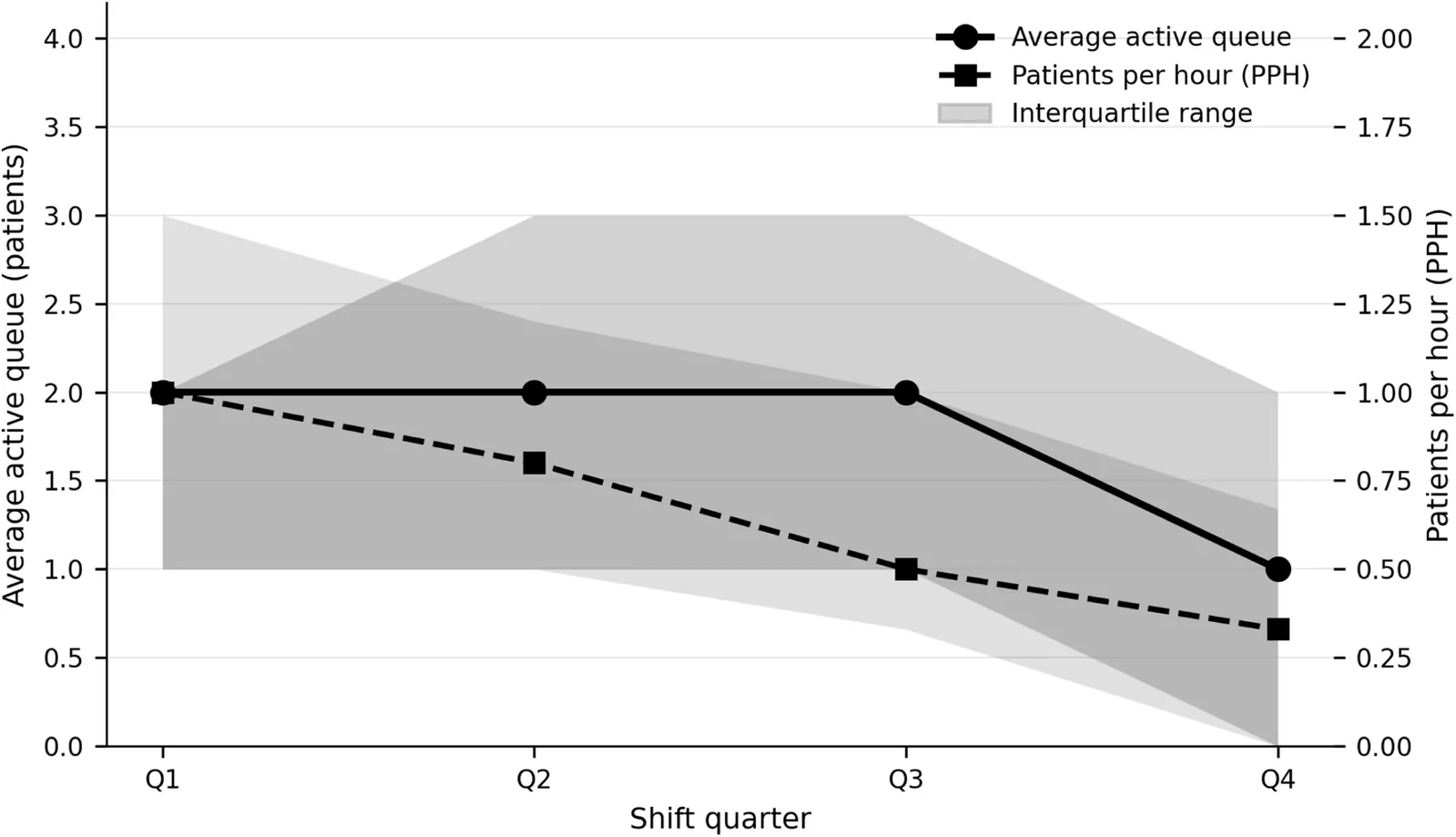
Median patients per hour (PPH) and median average active queue (AVQ) across shift quarters Q1–Q4 (n = 1 289 shifts). Shaded bands represent interquartile ranges.

The associations between AQ and EC flow metrics are shown in Fig. 3. A higher number of patients waiting at shift start was positively associated with AVQ (rho = 0.19, p < 0.001), while more new arrivals during the shift showed a paradoxical inverse association (rho = −0.16, p < 0.001). Mean total patients in the unit and mean boarders showed weak associations with AVQ (rho = 0.06 and −0.07 respectively) which, while statistically significant (both p < 0.05), are unlikely to be of clinical or operational relevance given their small effect sizes. High acuity patient proportion was strongly inversely associated with PPH (rho = −0.44, p < 0.001) but showed no meaningful association with AVQ (rho = 0.06, p = 0.029) or peak AQ (rho = −0.03, p = 0.308).

**Fig. 3:**
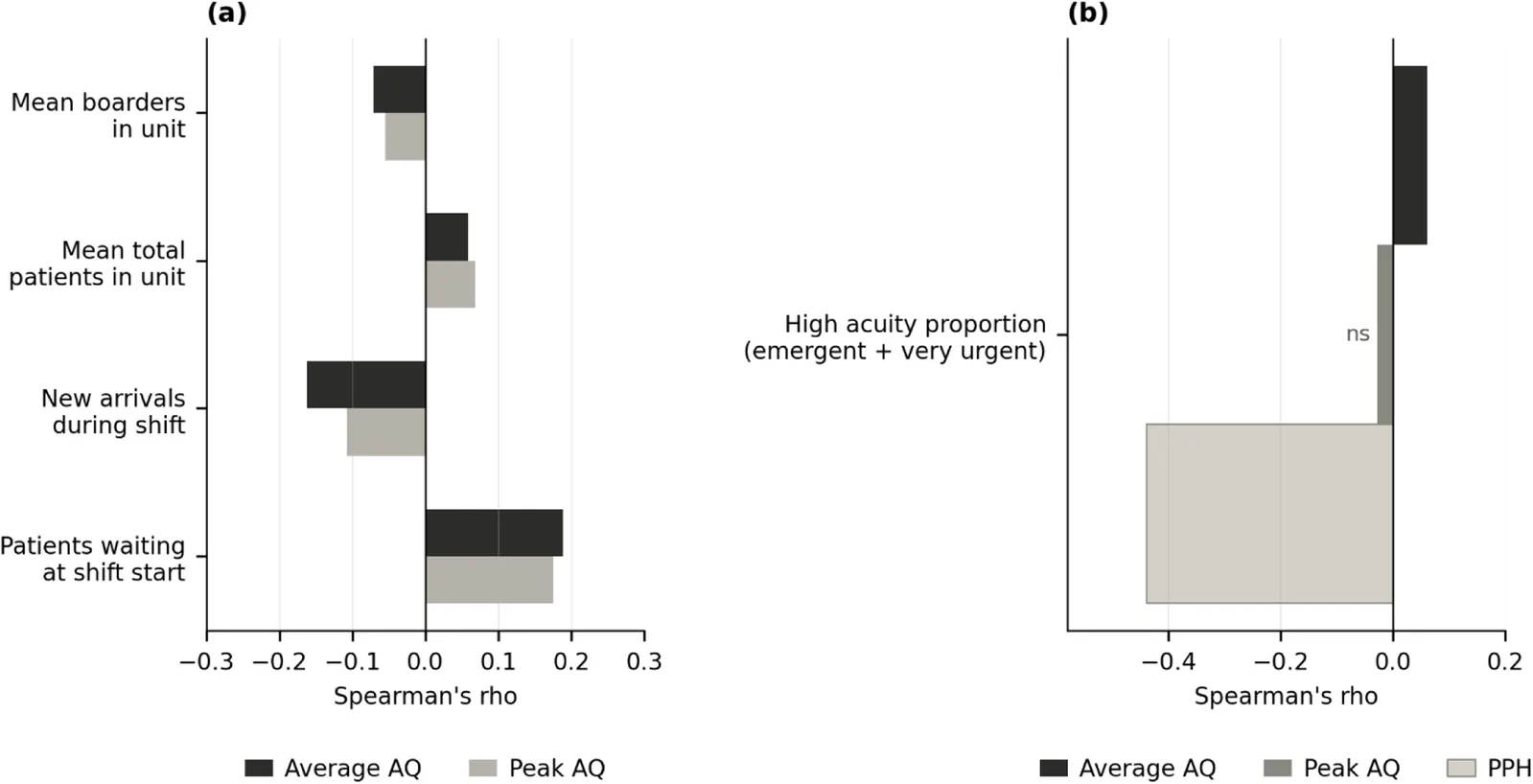
Spearman's rank correlations between emergency centre flow metrics and clinician activity measures (n = 1 289 shifts). (a) Associations between average and peak active queue (AQ) and four shift-level flow metrics. (b) Associations of high acuity patient proportion (emergent and very urgent triage categories) with AQ and patients per hour (PPH). ns = not significant (p = 0.308). All other associations p < 0.05.

## Discussion

This study aimed to describe the active patient queue at a district-level EC in Cape Town, South Africa, and to examine the factors associated with variation in queue size. Clinicians managed a median of 1.5 patients concurrently across the shift as a whole, with a stable mid-shift queue of 2.0 patients during the active working period, and a median peak of 3 patients at any single point. The queue remained stable across the first three shift quarters before clearing in the final quarter, while PPH declined progressively throughout the shift. These findings extend understanding of clinician workload beyond throughput measures alone and offer insight into how clinicians work, what drives queue accumulation, and what system-level factors might be targeted to optimise EC flow.

Emergency clinicians do not work sequentially. They are continually required to manage interruptions, task-switch, and prioritise competing demands as new patients arrive and existing cases evolve [13,14]. The active queue is therefore not a dysfunction but the normal operating state of EC clinical practice. Joseph et al. demonstrated a similar pattern in a multicentre HIC setting, with active queues increasing early in the shift, plateauing mid-shift, and declining toward shift end. It should be noted that Joseph et al. used a retrospective cohort design deriving AQ from electronic medical record timestamps, as did the current study. Both methods therefore share similar limitations regarding timestamp accuracy and documentation behaviour, and findings from both should be interpreted accordingly [9]. The two methods measure related but distinct constructs, and direct comparison of absolute queue sizes should be interpreted with this methodological difference in mind. Peak queue sizes in their study ranged between 5 and 7 patients, substantially higher than the median peak of 3 observed here. This discrepancy likely reflects the lower overall PPH at this EC, previously described by Hoffe et al. [8] and attributed to staffing category, resource constraints, and systems factors inherent to LMIC district-level care. The moderate positive correlation between AVQ and PPH (rho = 0.43) suggests that clinicians who see more patients per hour also carry a larger concurrent load, consistent with prior work demonstrating that experience and seniority are associated with both higher throughput and greater capacity to manage concurrent workload [8,[15], [16], [17]].

The variation in AVQ across clinician categories reflects the role of experience and clinical autonomy in shaping how concurrent care is managed. Locum doctors carried the highest median AVQ and peak AQ, likely reflecting greater independence and capacity to open and hold multiple consultations simultaneously. Interns carried the lowest AVQ on both measures, a finding consistent with the supervised practice model, where interns typically complete one consultation before initiating the next. Out-of-department clinicians showed similarly low AVQs despite also working outside their primary department. Unlike locums, who are typically experienced medical officers taking additional EC shifts with independence, out-of-department clinicians are rotated from other hospital departments, lack EC-specific training, and work under closer supervision. The divergence between these two groups reflects differences in EC experience and clinical autonomy rather than employment status alone.

The finding that high acuity patient proportion strongly suppressed PPH (rho = −0.44) but showed no meaningful association with AVQ (rho = 0.06) suggests that acuity constrains throughput without proportionally expanding the concurrent load. Experienced clinicians may absorb acuity-related complexity through improved task management without opening additional patients [8]. This association is descriptive and does not establish causality. A stable AVQ in high-acuity shifts may reflect constrained safe practice rather than an absence of workload effect, and prospective studies with direct observation are needed to disentangle these mechanisms.

The observed plateau in AVQ during mid-shift, despite ongoing new patient arrivals, is consistent with the presence of a workload ceiling, a point beyond which clinicians may be unable to expand their concurrent load regardless of demand. However, this plateau may equally reflect documentation behaviour, investigation delays, patient allocation practices, or shift-end routines, and should be interpreted as one plausible explanation rather than a definitive conclusion. The positive association between patients waiting at shift start and AVQ suggests that inherited backlog drives early queue accumulation, while the paradoxical inverse association between new arrivals and AVQ is consistent with surge adaptation behaviour, where under high-volume conditions, clinicians appear to prioritise faster turnover of lower-acuity cases, limiting the expansion of their open patient list. This aligns with prior findings of reduced PPH with increasing patient volume at this EC [8]. The rising AVQ with consecutive shift number from 1.50 on first shifts to 2.00 after more than six consecutive shifts may reflect the cumulative effect of fatigue or carryover complexity, and has implications for roster design and safe working hour limits that merit further investigation.

Nearly 2 out of 3 shifts (63.6%) ended with at least one patient still under active care, representing a handover burden. Joseph et al. reported similar findings, and prior work has demonstrated that signed-out patients negatively impact incoming clinician productivity [9,18]. It should be noted that Joseph et al. excluded the final hour of each shift from their analysis, as it was specifically designated for closing out patient encounters. This methodological difference may account for some of the variation in apparent handover burden between the two studies and should be considered when making direct comparisons. Reducing this burden likely requires addressing the upstream causes of prolonged queue occupancy, principally delays in investigation results and disposition decision-making, rather than simply adjusting shift boundaries. Point-of-care testing, streaming by acuity, and staggered shift starts with structured handover overlap are among the system-level interventions most likely to reduce AVQ and its associated handover burden in resource-constrained settings.

This study has several limitations. As this is a single-site retrospective, descriptive analysis, no causal inferences can be drawn and findings should be considered hypothesis-generating. Direct operational applicability to the current EC environment is further limited by the pre-pandemic nature of the dataset. However, it represents a well-characterised baseline against which post-pandemic comparisons can be made. AVQ calculations are dependent on accurate HECTIS timestamping, and documentation delays may introduce measurement bias. The absence of acuity adjustment at the individual clinician shift level means that AVQ differences between categories cannot be fully disentangled from case-mix effects. Several construct validity considerations should be acknowledged. AQ as derived from HECTIS captures the number of patient episodes formally opened by a clinician that have not yet reached a recorded disposition decision. It does not directly measure cognitive workload or care intensity, and the interval between timestamps may be prolonged by factors external to the clinician, including laboratory turnaround, imaging, specialist response, or bed availability, rather than reflecting active workload. AQ is also shaped by documentation behaviour, e.g., clinicians who batch their entries, document retrospectively, or delay closing episodes will appear to carry a larger queue than those who document in real time. Local workflow practices, such as pre-shift preparation or post-shift completion of records, may further influence AQ at shift boundaries. AQ should therefore be interpreted as a useful operational proxy for unresolved clinician-attributed episodes rather than a direct or validated measure of workload.

A further limitation is that AQ as derived from HECTIS reflects individual clinician-level attribution and does not capture the team-based nature of EC care. Interns may open consultations subsequently reviewed and disposed of by a supervising clinician, potentially distorting AVQ differences between categories. Resuscitation patients managed by teams may appear attributed to a single clinician in HECTIS, and pre-shift practices may further distort AQ at shift boundaries. These socio-technical aspects of HECTIS data entry should be considered when interpreting findings. Despite these limitations, this study provides the first description of active patient queue dynamics at a district-level EC, using a large, methodologically clean dataset from a well-characterised high-volume facility. Findings should be interpreted in the context of this specific setting and staffing model, and external validation in other district-level ECs is required before broader generalisation.

## Conclusion

Emergency clinicians routinely manage concurrent patient loads rather than sequential consultations, and the active queue is the normal operating state of EC practice. This study provides the first quantitative description of active patient queue dynamics in a district-level EC, demonstrating a consistent mid-shift workload plateau, a noteworthy shift-end handover burden, and meaningful variation by clinician category and employment type. AVQ complements PPH by capturing a dimension of workload that throughput measures alone cannot reflect. Understanding queue dynamics, how they accumulate, what sustains them, and how they clear, is essential to designing staffing models, optimising shift structures, and targeting the system-level bottlenecks that drive delays in disposition decisions in resource-constrained EC settings.

## Dissemination of results

The results were presented at AfCEM 2024 as a poster presentation and disseminated to the respective Emergency Centre and Hospital managers, as well as to the Faculty of Emergency Medicine Cape Town.

## Funding

This study was funded by the authors. During the preparation of this work the authors used Claude (Anthropic) to improve language clarity and readability. After using this tool, the authors reviewed and edited the content as needed and take full responsibility for the content of the publication.

## Declaration of competing interest

CH is an editor of the African Journal of Emergency Medicine. CH was not involved in the editorial workflow for this manuscript. The African Journal of Emergency Medicine applies a double-blinded process for all manuscript peer reviews. The authors declared no further conflicts of interest.
